# Independent accretion of TIM22 complex subunits in the animal and fungal lineages

**DOI:** 10.12688/f1000research.25904.1

**Published:** 2020-08-28

**Authors:** Sergio A. Muñoz-Gómez, Shannon N. Snyder, Samantha J. Montoya, Jeremy G. Wideman

**Affiliations:** 1 Biodesign Center for Mechanisms of Evolution, School of Life Sciences, Arizona State University, Tempe, AZ, 85287, USA

**Keywords:** TIM22 complex, Mitochondrial protein import, Mitochondrial evolution, Neutral evolution

## Abstract

**Background:** The mitochondrial protein import complexes arose early in eukaryogenesis. Most of the components of the protein import pathways predate the last eukaryotic common ancestor. For example, the carrier-insertase TIM22 complex comprises the widely conserved Tim22 channel core. However, the auxiliary components of fungal and animal TIM22 complexes are exceptions to this ancient conservation.

**Methods:** Using comparative genomics and phylogenetic approaches, we identified precisely when each TIM22 accretion occurred.

**Results:** In animals, we demonstrate that Tim29 and Tim10b arose early in the holozoan lineage. Tim29 predates the metazoan lineage being present in the animal sister lineages, choanoflagellate and filastereans, whereas the erroneously named Tim10b arose from a duplication of Tim9 at the base of metazoans. In fungi, we show that Tim54 has representatives present in every holomycotan lineage including microsporidians and fonticulids, whereas Tim18 and Tim12 appeared much later in fungal evolution. Specifically, Tim18 and Tim12 arose from duplications of Sdh3 and Tim10, respectively, early in the Saccharomycotina. Surprisingly, we show that Tim54 is distantly related to AGK suggesting that AGK and Tim54 are extremely divergent orthologues and the origin of AGK/Tim54 interaction with Tim22 predates the divergence of animals and fungi.

**Conclusions:** We argue that the evolutionary history of the TIM22 complex is best understood as the neutral structural divergence of an otherwise strongly functionally conserved protein complex. This view suggests that many of the differences in structure/subunit composition of multi-protein complexes are non-adaptive. Instead, most of the phylogenetic variation of functionally conserved molecular machines, which have been under stable selective pressures for vast phylogenetic spans, such as the TIM22 complex, is most likely the outcome of the interplay of random genetic drift and mutation pressure.

## Introduction

Mitochondria evolved from an ancient alphaproteobacterial endosymbiont ( Martijn *et al.,* 2018; Roger *et al.,* 2017). The integration of the symbiont into host cell processes required the evolution of dedicated machinery for the import of host-encoded proteins ( Roger *et al.,* 2017). The establishment of symbiont protein import allowed the transfer of many genes from the symbiont to the host genome as well as domestication of symbiont metabolic processes (e.g., via the evolution of mitochondrial carrier family proteins MCFs) ( Cavalier-Smith, 2006). Understanding how symbionts become organelles and integrate into host cell processes requires an understanding of how protein import machineries originate and diversify.

Proteins imported into the mitochondria require several dedicated protein complexes to ensure proper sorting and assembly into mitochondrial subcompartments, including the mitochondrial outer membrane (MOM), the mitochondrial inner membrane (MIM), the intermembrane space (IMS) and the matrix ( Wiedemann & Pfanner, 2017). The translocase of the mitochondrial outer membrane complex (TOM) facilitates the transport of all mitochondrial proteins (with the exception of some MOM proteins ( Becker *et al.,* 2008; Becker *et al.,* 2011; Dimmer *et al.,* 2012; Doan *et al.,* 2020)). The sorting and assembly machinery complex (SAM) is required for the import and assembly of MOM β-barrel proteins like Tom40, Sam50, Mdm10, and Porin. Two translocase of the mitochondrial inner membrane (TIM) complexes facilitate MIM protein import. The TIM23 complex is required for the membrane translocation of proteins with presequences that are directed into the matrix as well as single-pass transmembrane domain (TMD) proteins ( Mokranjac & Neupert, 2010). The TIM22 complex is responsible for inserting and assembling multi-pass TMD proteins like MCFs into the MIM ( Horten *et al.,* 2020).

All of these protein complexes are inferred to have been present in the last eukaryotic common ancestor (LECA) and the general phylogenetic profiles of their components have been recently reported ( Fukasawa *et al.,* 2017; Mani *et al.,* 2015). Investigations have so far focused on the broad distribution of subunits across eukaryotes, leaving some details unexplored, like the evolution of TIM22 complex components.

The TIM22 complex functions to assemble multi-pass TMD proteins like MCFs into the MIM ( Kerscher *et al.,* 1997). In both human and yeast cells, TIM22 substrates first cross the MOM via TOM and are delivered to the small tim complexes (Tim9-10 and Tim8-13 complexes; Tim12 is additionally present in *S. cerevisiae*, whereas Tim10a and Tim10b, as well as Tim8a and Tim8b are present in human) which shuttle proteins through the IMS to TIM22 ( Beverly *et al.,* 2008; Davis *et al.,* 2007; Gentle *et al.,* 2007; Hoppins & Nargang, 2004; Koehler *et al.,* 1998; Sirrenberg *et al.,* 1998).

 In *S. cerevisiae*, the TIM22 complex comprises Tim22, Tim54, Tim12, Tim18, and Sdh3, whereas in human, the complex contains Tim22, Tim29, AGK (acyglycerol kinase), and a subset of small Tims (Tim9, Tim10a, and Tim10b); as the small Tims are soluble proteins that shuttle hydrophobic substrates, they might not constitute stable components of the TIM22 complex. In *S. cerevisiae*, Tim54 plays a role in TIM22 complex stability as well as assembly of the Yme1 complex ( Hwang *et al.,* 2007; Kerscher *et al.,* 1997). Sdh3 (a component of succinate dehydrogenase [SDH] – Complex II of the electron transport chain) and Tim18 (a paralogue of Sdh4) facilitate the integration of Tim54 into the TIM22 complex ( Gebert *et al.,* 2011; Kerscher *et al.,* 2000; Koehler *et al.,* 2000). A recent cryo-EM structure has been solved for the human TIM22 complex, showing how the components interact within the MIM ( Qi *et al.,* 2019). In human mitochondria, Tim29 stabilizes the TIM22 complex and links it to TOM ( Callegari *et al.,* 2016; Kang *et al.,* 2016). AGK plays a role in the function of TIM22 as well as lipid metabolism ( Kang *et al.,* 2017; Vukotic *et al.,* 2017).

Because animal and fungal TIM22 complexes are best characterized, both structurally and functionally, these lineages offer an ideal case to dissect the fine-grained evolutionary history of multi-protein complexes. Apart from the central Tim22 subunit ( Fukasawa *et al.,* 2017; Žárský & Doležal, 2016), the origin and evolution of the TIM22 complex components in animals and fungi has not been extensively investigated. In this paper, we explore the evolutionary history of the TIM22 complex in animals and fungi, using homology searching and phylogenetic methods. We found that each lineage’s TIM22 complex accreted subunits independently. We place our findings in a larger theoretical framework recently developed by Lynch ( Lynch, 2020; Lynch & Trickovic, 2020). We argue that most of the structural variation seen in the functionally conserved TIM22 complex across the Holozoa and the Holomycota is non-adaptive. The evolutionary history of the TIM22 complex probably represents an example of effectively neutral divergence from an optimal mean phenotype which has primarily been governed by the joint forces of drift and mutation.

## Results and discussion

### Tim29, AGK, Tim10b, and Tim8b have varied phyletic distribution in the holozoan lineage

Recent investigations have identified complex components beyond Tim22 in human cells, namely Tim29 and AGK ( Callegari *et al.,* 2016; Kang *et al.,* 2016; Kang *et al.,* 2017; Mårtensson & Becker 2017; Qi *et al.,* 2019; Vukotic *et al.,* 2017). Tim29 was originally identified as a complex component primarily involved in complex assembly and stability ( Kang *et al.,* 2016) but was subsequently also shown to be important for protein import ( Callegari *et al.,* 2016). The Sengers syndrome-associated AGK was originally shown to catalyze the phosphorylation of acylglycerols to lysophosphatidic and phosphatidic acid in the MIM ( Bektas *et al.,* 2005), but has recently been identified as a TIM22 complex member in human cells ( Kang *et al.,* 2017; Vukotic *et al.,* 2017). A cryo-EM structure that includes Tim22, Tim29, AGK, and the small Tims (Tim9, Tim10a, and Tim10b) has recently been reported ( Qi *et al.,* 2019). Since Tim29, AGK, and Tim10b are not present in fungi ( Fukasawa *et al.,* 2017), we sought to determine when in the holozoan lineage these proteins were gained.

**Figure 1.**
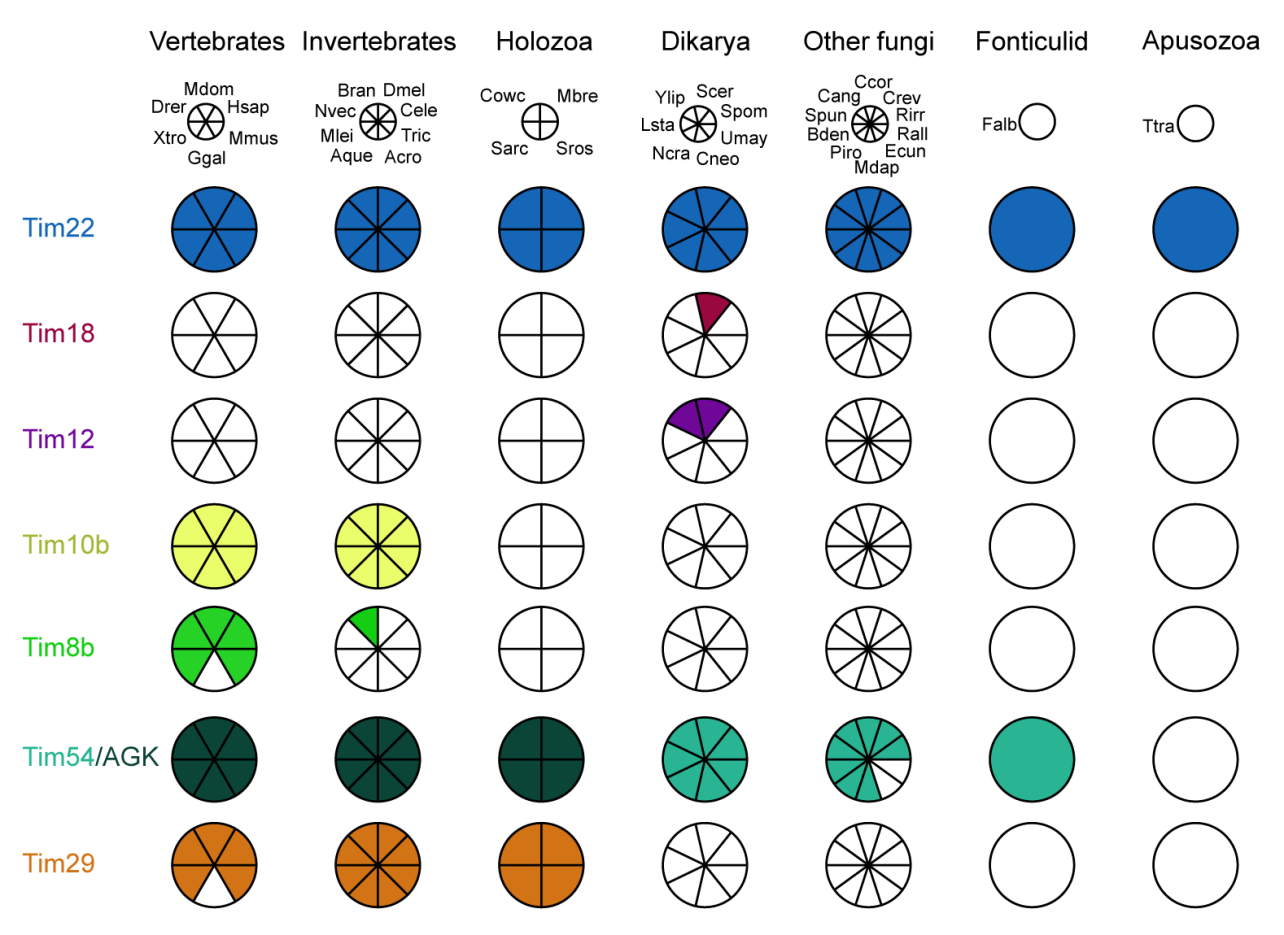
Distribution of TIM22 complex components across eukaryotes. The Coulson plot was generated using the Coulson Plot Generator ( Field *et al.,* 2013). Abbreviations: **Vertebrates**: Hsap, *Homo sapiens*; Mdom, *Monodelphis domesticus*; Drer, *Danio rerio*; Xtro, *Xenopus tropicalis*; Ggal, *Gallus gallus*; Mmus, *Mus musculus*; Invertebrates: Dmel, *Drosophila melanogaster*; Cele, *Caenorhabditis elegans*; ; Tric, *Trichoplax sp*.; Acro, *Acropora sp.*; Aque, *Amphimedon queenslandica*; Mlei, *Mnemiopsis leidyi*; Nvec, *Nematostella vectensis*; Bran, *Branchiostoma* sp.; **Unicellular Holozoa**: Mbre, *Monosiga brevicollis*; Cowc, *Capsaspora owczarzaki*; Sarc, *Sphaeroforma arctica*; Sros, *Salpingoeca rosetta*; **Fungi**: Spom, *Schizosaccharomyces pombe*; Scer, *Saccharomyces cerevisiae*; Ncra, *Neurospora crassa*; Cneo, *Cryptococcus neoformans*; Umay, *Ustilago maydis*; Lsta, *Lipomyces starkeyi*; Ylip, *Yarrowia lipolytica*, Bden, *Batrachochytrium dendrobatidis*; Mdap, *Mitosporidium daphnia*; Ecun, *Encephalitozoon cuniculi*; Piro, *Piromyces* sp.; Spun, *Spizellomyces punctatus*; Rirr, *Rhizophagus irregularis*; Crev, *Coemansia reversa*; Ccor, *Conidiobolus coronatus*; Cang, *Catenaria anguillulae*; Rall, *Rozella allomyces*; **Fonticulids**: *Fonticula alba*; **Apusozoa**: Ttra, *Thecamonas trahens*.

Using the reciprocal best BLAST search method, we identified orthologues of Tim29 in the genomes of most animal species and unicellular eukaryotes (i.e., protists) most closely related to animals ( Figure 1). This means Tim29 originated prior to the origin of animals. We could not identify Tim29 in *Gallus gallus*; however, orthologous sequences were recovered from other birds and reptiles, suggesting loss of Tim29 is limited to chickens. We used our set of Tim29 sequences to search for orthologues across eukaryotes using the HMMer server ( Finn *et al.,* 2011) at EBI. When restricting our taxon searching to exclude holozoans, we retrieved no hits below our 0.01 e-value significance cut off, strongly suggesting that no homologues of Tim29 exist outside Holozoa ( *Extended data*, Supplemental Text File 1; Wideman *et al*., 2020).

To determine when in the holozoan lineages the TIM22 subunits AGK, Tim10b, and Tim8b first appeared, we collected sequences related to AGK and the small Tims from diverse holozoan genomes. We aligned sequences using MUSCLE ( Edgar, 2004) and performed phylogenetic reconstructions using RAxML ( Stamatakis, 2014) and MrBayes ( Ronquist *et al.,* 2012).

**Figure 2.**
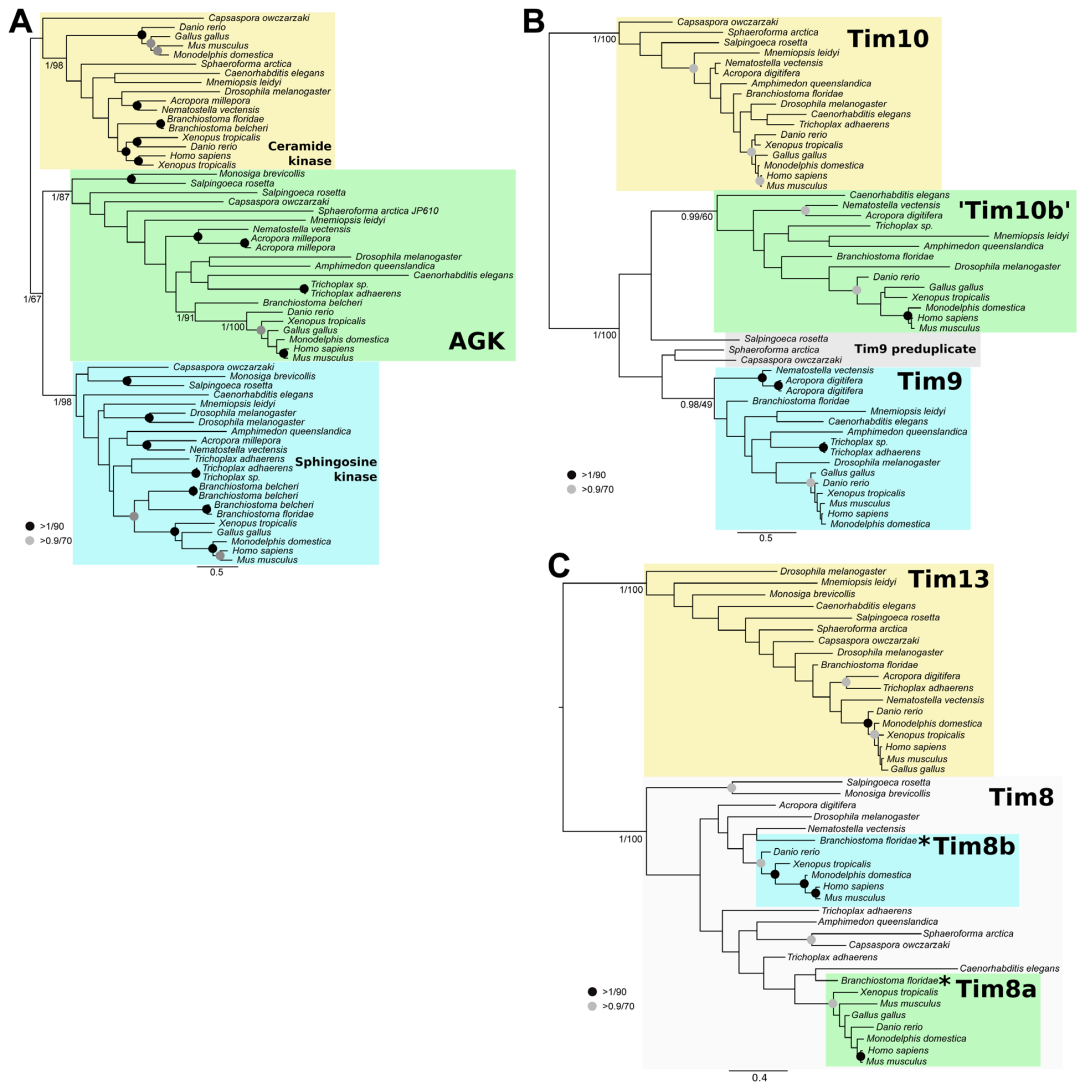
Phylogenetic analysis of holozoan TIM22 subunits. ( **A**) Acylgylcerol Kinase (AGK) was present in the ancestral holozoan. ( **B**) Tim10b arose from a duplication of Tim9 at the base of metazoans. ( **C**) Tim8a and Tim8b arose from a duplication of Tim8 at the base of chordates. Homologues of AGK and small tims were collected from a subset of holozoan taxa using BLASTp. Sequences were aligned and trimmed resulting in 296, 75, and 73 sites for AGK, Tim9/10, and Tim8/13, respectively. The asterisks indicate true Tim8a and Tim8b orthologues that are not supported in our tree but are confirmed by best BLAST analysis. The resulting alignments were subjected to phylogenetic analysis using MrBayes ( Ronquist *et al.,* 2012) for posterior probability and RAxML ( Stamatakis, 2014) for maximum likelihood frameworks.

The phylogenetic reconstruction of AGK and related sequences clearly distinguish putative clades of holozoan AGK, ceramide kinase, and sphingosine kinase indicative of a pre-metazoan ancestry of these enzymes ( Figure 2A). We did not include representatives from an outgroup as the best BLAST hits of AGK outside the holozoan lineage were cyanobacteria, oomycetes, and plants, suggesting that a detailed analysis of this gene family is required to understand its origin and evolutionary history in eukaryotes.

For the small Tims, the reconstructed phylogenies ( Figures 2B and 2C) include well-supported Tim10a and Tim13 clades. This suggests that Tim10b was the result of a duplication of Tim9 at the base of animals ( Figure 2B), whereas Tim8a and Tim8b are the result of a duplication at the base of chordates ( Figure 2C). Although we did not recover Tim8a and Tim8b sequences from *Branchiostoma floridae* within the vertebrate clades of Tim8a and Tim8b ( Figure 2C, asterisks), their best BLAST hits are clearly Tim8a and Tim8b from vertebrates, respectively. We therefore conclude that Tim8a and Tim8b arose prior to the divergence of chordates from the rest of animals. We were unable to recover small Tim sequences from the Choanoflagellate *Monosiga brevicollis*, but this is likely due to an incomplete database as Tim9 and Tim10 are probably essential in holozoans.

These results demonstrate that TIM22 complex subunits were accreted very early in the holozoa. Tim29 and AGK (but see below) appear to be gained shortly after the holozoan lineage diverged from the holomycotan lineage, Tim10b originated shortly after the origin of animals, and Tim8b originated after the origin of chordates. This means that Tim29 and AGK predate the origin of animals and have persisted in this lineage for about a billion years and Tim10b arose shortly thereafter.

### Tim54 is related to AGK and diverged at the base of fungi whereas Tim18 and Tim12 are the result of Saccharomycotina-specific gene duplications

Components of the TIM22 complex were identified in fungi much earlier than the recent discoveries in animals ( Kerscher *et al.,* 1997; Kerscher *et al.,* 2000; Koehler *et al.,* 2000). Tim54 appears to be involved in complex stability as well as integration of MIM proteases ( Hwang *et al.,* 2007; Kerscher *et al.,* 1997), but its mechanism of action is unclear. In yeast, Tim18 is related to Sdh4 of the SDH complex ( Kerscher *et al.,* 2000; Koehler *et al.,* 2000). Another SDH complex member, Sdh3 interacts with Tim18 as a TIM22 complex module that is integrated into the MIM by the OXA complex ( Stiller *et al.,* 2016). Sdh3 and Tim18 are involved in the biogenesis and assembly of Tim22 and Tim54 into a functional TIM22 complex ( Gebert *et al.,* 2011). Finally, Tim12 is a small Tim that is not found outside the yeast lineage.

Using the reciprocal best BLAST method, we were able to identify Tim54 in representatives from every major fungal lineage as well as *Fonticula alba*, but no other eukaryotic lineage ( Figure 1). We were unable to identify Tim54 in *Rozella allomycis* or microsporidians except *Mitosporidium daphniae*, a short-branching microsporidian with canonical mitochondria ( Haag *et al.,* 2014). We used our set of Tim54 sequences to search for orthologues across eukaryotes using the HMMer ( Finn *et al.,* 2011) server at EBI. When restricting our taxon searching to exclude fungi and *Fonticula*, we surprisingly retrieved AGK sequences from animals (141 hits above threshold) and *Capsaspora* as top hits ( *Extended data*, Supplemental Text File 2; Wideman *et al*., 2020). These results indicate that Tim54 and AGK likely share a common ancestor; however, the diacylglycerol kinase (DAGK) domain is now virtually undetectable in fungal sequences.

**Figure 3.**
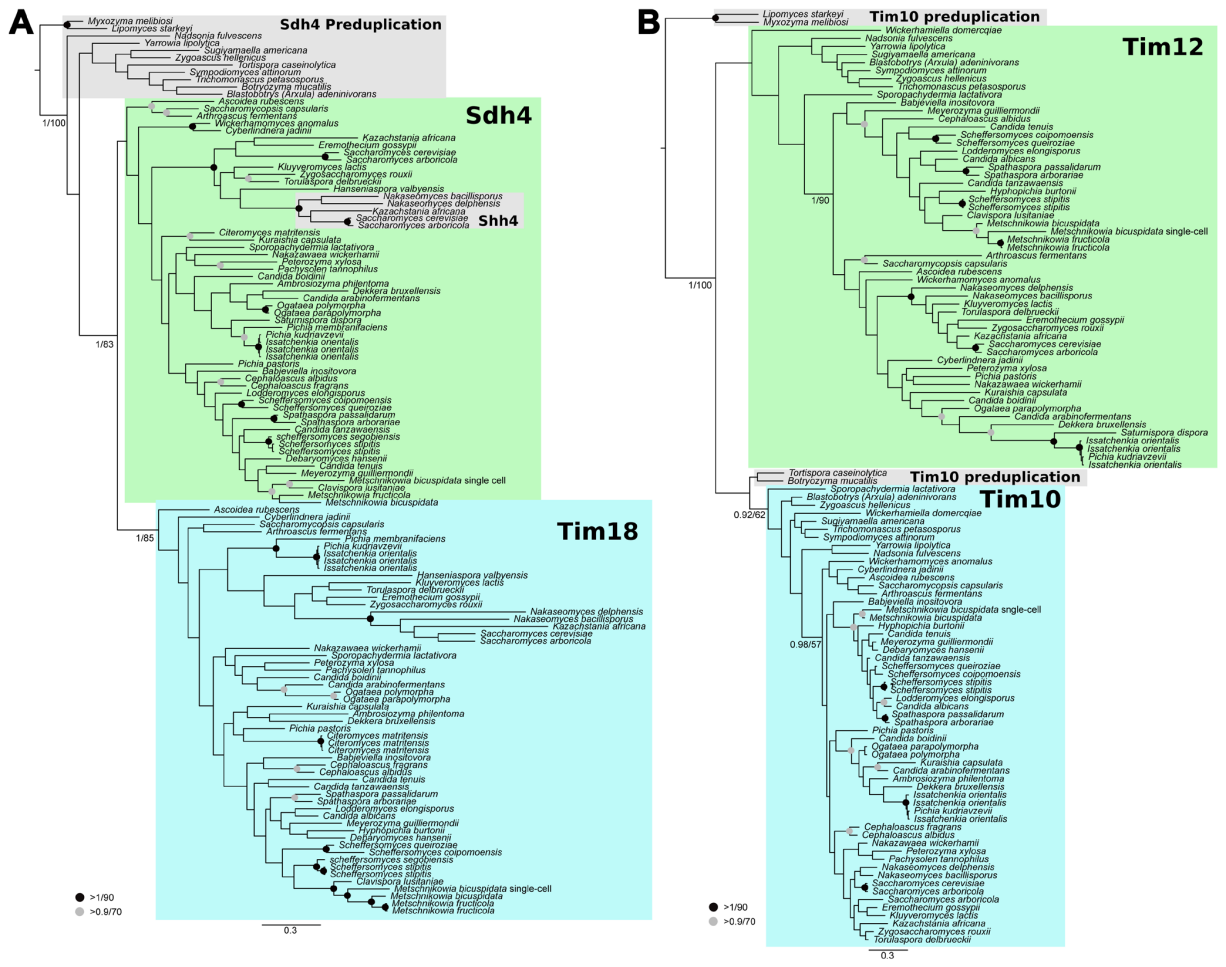
Phylogenetic analysis of Saccharomycotina TIM22 subunits. ( **A**) Tim18 arose from a duplication of Sdh4 deep within the yeast lineage. ( **B**) Tim12 arose from a duplication of Tim10 near the base of Saccharomycotina. Homologues of Tim18/Sdh4 and Tim10/12 were collected from sequenced Saccharomycotina on Mycocosm ( Grigoriev *et al.,* 2013) using BLASTp. Sequences were aligned and trimmed resulting in 128 and 82 sites for Tim18 and small tims, respectively. The resulting alignments were subjected to phylogenetic analysis using MrBayes ( Ronquist *et al.,*2012) for posterior probability and RAxML ( Stamatakis, 2014) for maximum likelihood frameworks.

To determine when Tim18 and Tim12 originated, sequences related to Tim18, Sdh4, Tim10, and Tim12 were collected from all Saccharomycotina in the Mycocosm database ( Grigoriev *et al.,* 2013). We trimmed long sequences and removed any spurious hits (as determined by reciprocal BLAST into the *S. cerevisiae* S288c genome). Sequences were aligned using MUSCLE ( Edgar, 2004), manually trimmed, and phylogenies reconstruction using RAxML ( Stamatakis, 2014) and MrBayes ( Ronquist *et al.,* 2012) for likelihood and posterior probability calculations, respectively. The phylogenetic reconstruction of Tim18 indicates that a duplication occurred after the divergence of early-branching Saccharomycotina (e.g. *Lipomyces* and *Yarrowia*), but before the divergence of a major clade that includes *Wickerhamomyces* and *Saccharomyces* ( Figure 3A). An additional Sdh4 paralogue Shh4 is the result of the more recent Saccharomyces lineage whole genome duplication/hybridization event ( Kellis *et al.,* 2004; Marcet-Houben & Gabaldón, 2015; Wolfe & Shields, 1997). The phylogenetic reconstruction of Tim12 suggests that a duplication of the Tim10 protein occurred even earlier in Saccharomycotina ( Figure 3B) as only the earliest-branching species lack clear Tim12 representatives (e.g., *Lipomyces starkeyi*).

**Figure 4.**
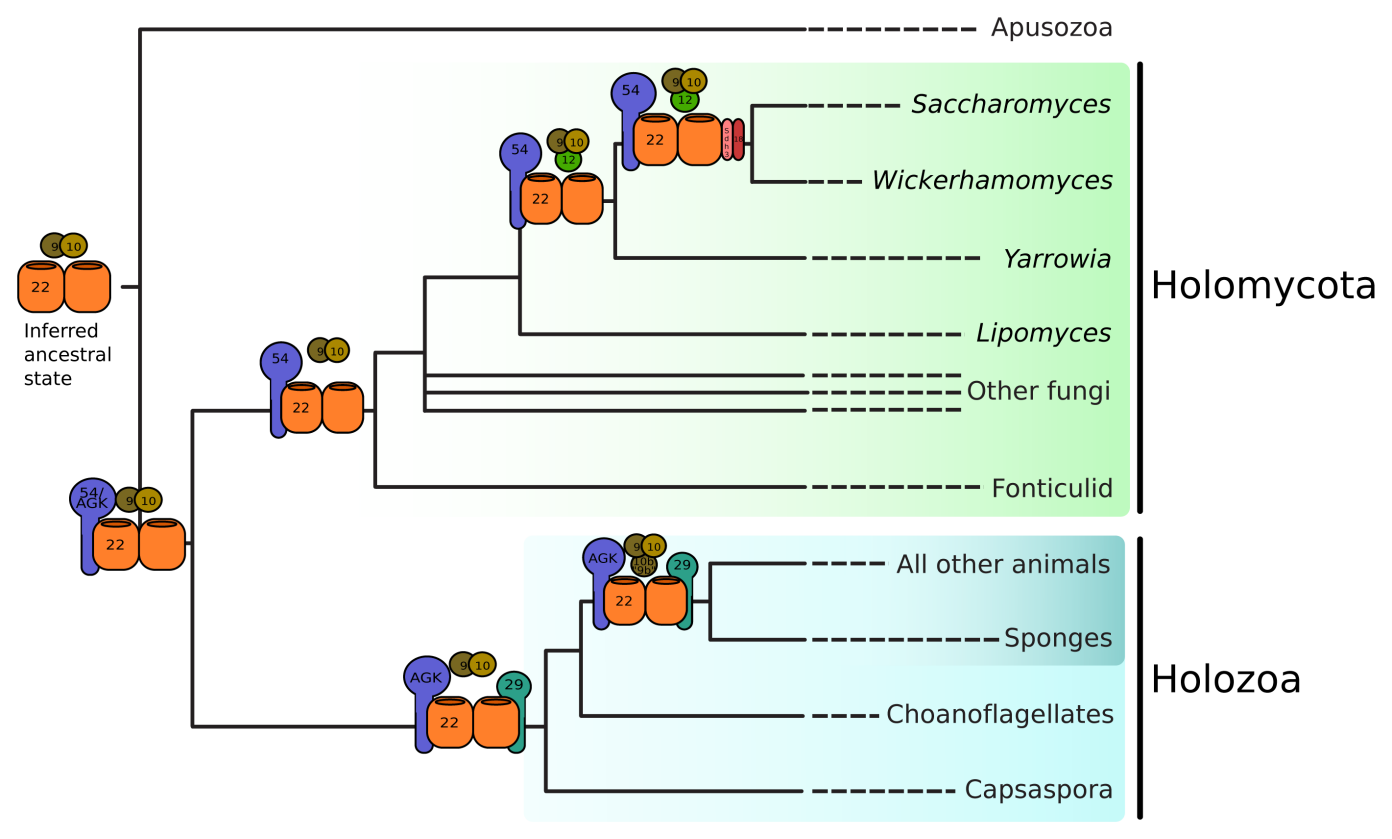
TIM22 complex subunits were gained independently in the animal and fungal lineages. The ancestral TIM22 complex comprises a single subunit, Tim22, which likely interacted with the small Tims, Tim9 and Tim10. The ancestral opisthokont TIM22 complex contained an additional subunit, AGK, which retains a diacylglycerol kinase (DAGK) domain in holozoans. In the holomycotan lineage, the DAGK domain was lost or diverged beyond recognition, but stabilized into Tim54, which is well-conserved across Holomycota. Early in the evolution of Saccharomycotina, Tim12 was gained via a duplication of Tim10, and Tim18 was gained via a duplication of Sdh4. Shortly after the divergence of Holozoa from Holomycota, Tim29 was gained as a subunit of the TIM22 complex. Tim10b was gained after the divergence of Metazoa from the unicellular holozoans from a duplication of Tim9. Tim8a and Tim8b result from a duplication of Tim8 in the lineage leading to chordates (not shown).

In contrast to the animal TIM22 complex, which accreted subunits early in the evolution of animals, we demonstrate that a gradual accretion of TIM22 complex subunits occurred in the lineage leading to *S. cerevisiae*. Tim54 is likely a divergent fungal AGK which lost the DAGK domain after the divergence of holomycota from holozoa ( Figure 4). Tim18 and Tim12 are respectively derived from duplications of Sdh4 and Tim10 deep within the Saccharomycotina. It is unknown if other fungal lineages have undergone similar expansions of the TIM22 complex.

## Conclusions

The first elements of a larger theoretical framework to quantitatively understand the macroevolution of cells, their organelles, and molecular machines have been developed ( Lynch, 2020; Lynch & Trickovic, 2020). Our findings are consistent with the predictions made by the theory of effectively neutral divergence of mean phenotypes across major phylogenetic lineages ( Lynch, 2020). This theory assumes that the selective pressures on many molecular machines have remained relatively constant for long stretches of macroevolutionary time. This is most likely the case for many multi-protein complexes whose functions are strongly conserved across vast phylogenetic spans. Mitochondrial protein import complexes, such as TIM22, are good examples of such strongly functionally conserved systems. The TIM22 complex has to physically interact with dozens or even hundreds of substrates for their proper insertion into the MIM. This implies that its functional divergence is constrained (and/or buffered) by its many physical interactions. However, slight deviations from an optimal (functional) phenotypic mean could be expected as a consequence of a permissive population-genetic environment, i.e., stronger drift due to historical bottlenecks or smaller effective population sizes. As selective pressures are assumed to remain constant for many conserved multi-protein complexes in stable cellular environments, most phenotypic divergence would be dictated by the combined action of random genetic drift and mutation pressure. Most divergence seen in multi-protein complexes or molecular machines, therefore, would primarily be non-functional and non-adaptive but mostly structural (e.g., subunit composition) in character.

Many other observations appear to be compatible with this view. The common recruitment of paralogous subunits, as well as the presence of highly derived and lineage-specific subunits in multi-protein complexes provide general examples. More specifically, kinetoplastids offer another example in a protein-import complex. *Trypanosoma brucei* lacks the TIM23 complex and instead contains a bifunctional Tim22 protein that acts both as a presequence and a carrier translocase complex ( Harsman *et al.,* 2016; Schneider, 2020; Singha *et al.,* 2008). We have recently suggested that the bifunctional Tim22 complex of kinetoplastids evolved neutrally via homologue replacement of ancestral components followed by loss of TIM23 ( von Känel *et al.,* 2020). This massive divergence from the ancestral state probably added no obvious benefit to the organism. As long as the whole still performs the same function, it doesn’t matter what parts are used. This is also the sentiment behind neutral evolution of cellular structures ( Wideman *et al.,* 2019; Zhang, 2018). Evolutionary divergence primarily dictated by drift and mutation (under stable selective pressure in constant cellular environments ( Lynch, 2020)); would certainly allow for the constructive, ratchet-like, structural evolution of multi-protein complex (( Gray *et al.,* 2010; Stoltzfus, 1999; Stoltzfus, 2012; Wideman *et al.,* 2019); a.k.a., constructive neutral evolution). Further comparative investigations at the bench are required to determine what functional differences exist between fungal and animal TIM22 complexes, and whether other eukaryotic lineages have accreted subunits in a similar way to opisthokonts ( Figure 4).

## Methods

### Homology searching

Tim22 orthologues were identified previously ( Žárský & Doležal, 2016). We used the reciprocal best hit method to identify Tim54 and Tim29 orthologues in fungi and holozoans, respectively. Briefly, *S. cerevisiae* Tim54 and *Homo sapiens* Tim29 were used as BLASTp ( Altschul *et al.,* 1997) queries into opisthokont predicted proteomes (see Figure 1 for organism list) using the NCBI BLAST server or Mycocosm database ( Grigoriev *et al.,* 2013). The top hit was retrieved and used as a BLASTp query into *S. cerevisiae* (for Tim54) or *H. sapiens* (for Tim29) proteome. If the top hit was the original BLASTp query, then the retrieved protein was considered orthologous. Using HMMer ( Finn *et al.,* 2011) at EBI we used our collected Tim54 and Tim29 sequences to build Hidden Markov Model profiles to identify orthologues across eukaryotes. Orthologue distribution across opisthokonts was visualized using the Coulson Plot Generator ( Field *et al.,* 2013).

### Phylogenetic analysis of AGK, Tim18, and small Tim homologues

Homologues of AGK, and small Tims were collected from metazoan predicted proteomes using BLASTp. Homologues of Tim18, Sdh4, Tim10, and Tim12 were collected from all sequenced Saccharomycotina from the Mycocosm database using BLASTp ( Grigoriev *et al.,* 2013). Pertinent homologues were aligned with MUSCLE ( Edgar, 2004) and manually trimmed using Mesquite v.2.75. Phylogenetic tree reconstructions were performed using MrBayes v.3.2.6 for Bayesian analysis ( Ronquist *et al.,* 2012). MrBayes analyses were run with the following parameters: prset aamodelpr = fixed (WAG); mcmcngen = 2,000,000; samplefreq = 1000; nchains = 4; startingtree = random; sumt burnin = 250. Split frequencies were checked to ensure convergence. Maximum-likelihood bootstrap values (100 pseudoreplicates) were obtained using RAxML v.8.2.10 ( Stamatakis, 2014) under the LG model ( Le & Gascuel, 2008).

## Data availability

### Source data

Sequence accession are provided in *Extended data*, Supplemental Table 1 ( Wideman *et al*., 2020). Sequences are from the NCBI database, the Mycocosm database at the Joint Genome Institute, or from the *Mnemiopsis leydii* genome database.

### Extended data

Figshare: Extended Data: Independent accretion of TIM22 complex subunits in the animal and fungal lineages. https://doi.org/10.6084/m9.figshare.12818786.v1 ( Wideman *et al*., 2020).

This project contains the following extended data:

- Supplemental Table 1. TIM22 complex subunit accessions collected in this investigation.
- Supplemental Text File 1. Tim29 HMMer results .txt file.
- Supplemental Text File 2. Tim54 HMMer results .txt file.
