# Supplementary material for: Independent accretion of TIM22 complex subunits in the animal and fungal lineages
Source: F1000Res. 2020 Aug 28;9:1060. [Version 1] doi: 10.12688/f1000research.25904.1 (PMC7523481; doi:10.12688/f1000research.25904.1)
Supplement: Peer review history

# Peer review: Reviewer response for version 1

**DOI:** 10.5256/f1000research.28588.r70541

**Published:** 2020-09-28

**Authors:** Thomas Becker

**Affiliations:** 1 Institute for Biochemistry and Molecular Biology, University of Freiburg, Freiburg, Germany

The TIM22 complex promotes import of carrier proteins into the inner mitochondrial membrane. Recent studies showed the composition of the TIM22 complex in human mitochondria, which displayed remarkable differences to the yeast homolog. Munoz-Gomez and colleagues investigates the phylogenetic origin of the TIM22 subunits. The authors found that yeast Tim54 and human AGK are distantly related. The furthermore showed that yeast Tim12 and Tim18 arose from gene duplication of Tim10 and Sdh3, respectively. Tim29 is specific for holozoan, while Tim12, Sdh3, Tim18 are specific for holomycota. Overall, the findings are interesting and well presented. I have only minor recommendations.

 The authors should describe in more detail the substrates of the carrier pathway, including substrates with an uneven number of transmembrane spans that have been reported recently (Gomkale et al., 2020 ^1^; Rampelt et al., 2020 ^2^).

 The authors should name all import pathways into mitochondria in the introduction. The current version is rather incomplete and lacks the MIM-dependent import pathway into the outer membrane. Furthermore, appropriate citations for TOM and SAM complexes should be added.

 The section about the TIM22 components should be modified. The authors should describe that Tim18/Sdh3 are important for the stability of the TIM22 complex. Tim54 could serve as a docking site for small TIM chaperones of the intermembrane space.

Is the work clearly and accurately presented and does it cite the current literature?

Yes

If applicable, is the statistical analysis and its interpretation appropriate?

I cannot comment. A qualified statistician is required.

Are all the source data underlying the results available to ensure full reproducibility?

Yes

Is the study design appropriate and is the work technically sound?

Yes

Are the conclusions drawn adequately supported by the results?

Yes

Are sufficient details of methods and analysis provided to allow replication by others?

Yes

Reviewer Expertise:

NA

I confirm that I have read this submission and believe that I have an appropriate level of expertise to confirm that it is of an acceptable scientific standard.

## References

[1] : Defining the Substrate Spectrum of the TIM22 Complex Identifies Pyruvate Carrier Subunits as Unconventional Cargos. *Curr Biol*.2020;30(6) : 10.1016/j.cub.2020.01.024 1119-1127.e5 10.1016/j.cub.2020.01.024 32142709PMC7090383

[2] : The mitochondrial carrier pathway transports non-canonical substrates with an odd number of transmembrane segments. *BMC Biology*.2020;18(1) : 10.1186/s12915-019-0733-6 10.1186/s12915-019-0733-6 31907035PMC6945462

# Peer review: Reviewer response for version 1

**DOI:** 10.5256/f1000research.28588.r71396

**Published:** 2020-09-24

**Authors:** Kevin R. Lynch

**Affiliations:** 1 Department of Pharmacology, University of Virginia, Charlottesville, VA, USA

The well-studied *S. cerevisiae* and human Tim22 complexes both function in the transport/insertion of integral membrane proteins into the inner mitochondrial membrane, yet the only conserved protein of the complexes is Tim22 itself. Through a variety of protein sequence comparison techniques, the authors document the appearance of various Tim22 complex proteins in holozoan and holomycotan lineages.  Surprisingly, the authors found that the animal Tim22 complex protein, AGK, is distantly related to the fungal Tim22 complex protein, Tim54. I do not have particular expertise in Molecular Evolution, rather my interest in the study is primarily through the narrower lens of an ongoing interest in the biochemistry of AGK.

 The authors do not state what regions of AGK and Tim54 were found to be related, and this information is of interest, because even when comparing AGK with the closely related sphingolipid kinases, the only similarity (detected by BLASTP searches) is in the amino terminal, ATP-binding region (identified by inference from human SPHK1 crystal structure). The authors report that AGK orthologs are readily identifiable when searching metazoan databases and can likewise be identified in a few unicellular eukaryotes that are closely related to animals ( *e.g.* choanoflagelattes, *Capsaspora*). This is readily verified with simple BLASTP searches using human AGK as a query sequence. However, after reading the paper twice, I remain confused as to whether AGK orthologs have been identified in other protist databases. Specifically, how do the authors distinguish between AGK orthologs and the paralogous DAG kinases? 

 While it is tangential to the subject of this paper, I note that AGK-focused publications have a somewhat tortuous history (of which the authors are, understandably, probably unaware). As documented in their Figure 2A, AGK is most closely related to sphingolipid kinases. That group in turn is more distantly related to the diacylglycerol kinase (DAGK) family and the NAD ^+^ kinases. The earliest AGK literature focused on its putative kinase activity. The authors reference the 2005 Bektas *et al*. paper in support of the contention that AGK is an acylglycerol kinase as is the common practice in AGK papers since 2005. However, there is little in the Bektas paper or elsewhere to validate this claim. Indeed, AGK appeared in the literature originally as a multi-substrate lipid kinase (PMID: 15252046 ^1^) and later as a possible ceramide kinase in *Drosophila* (PMID: 22069480 ^2^). Another group reported a failure to detect any kinase activity, i.e. AGK as an “orphan” kinase (PMID: 16269826 ^3^). I recommend that the authors consider citing all of these mutually contradictory reports since all save one have been neglected in the subsequent AGK literature – for no apparent reason except ‘everybody else does it’. The more recent AGK literature includes the discovery that humans born deficient in the protein suffer from Sengers syndrome, which is characterized by mitochondrial insufficiency. Subsequent to that discovery, AGK was found to be a component of the Tim22 complex in human cells. Interestingly, a single amino change (G126E) in human AGK that would eliminate enzyme activity (by inference from the better characterized sphingosine kinases) does not inhibit the import function of the Tim22 complex in cultured cells.

Is the work clearly and accurately presented and does it cite the current literature?

Partly

If applicable, is the statistical analysis and its interpretation appropriate?

Yes

Are all the source data underlying the results available to ensure full reproducibility?

Yes

Is the study design appropriate and is the work technically sound?

Yes

Are the conclusions drawn adequately supported by the results?

Partly

Are sufficient details of methods and analysis provided to allow replication by others?

Yes

Reviewer Expertise:

Sphingolipid biochemistry, specifically sphingosine-1-phosphate synthesis, transport, degradation and receptor binding.  Thus, as stated in my comments, my focus on AGK, which is a member of the sphingolipid kinase family.

I confirm that I have read this submission and believe that I have an appropriate level of expertise to confirm that it is of an acceptable scientific standard.

## References

[1] : MuLK, a eukaryotic multi-substrate lipid kinase. *J Biol Chem*.2004;279(37) : 10.1074/jbc.M405932200 38228-35 10.1074/jbc.M405932200 15252046

[2] : A suppressor/enhancer screen in Drosophila reveals a role for wnt-mediated lipid metabolism in primordial germ cell migration. *PLoS One*.2011;6(11) : 10.1371/journal.pone.0026993 e26993 10.1371/journal.pone.0026993 22069480PMC3206050

[3] : Further characterization of mammalian ceramide kinase: substrate delivery and (stereo)specificity, tissue distribution, and subcellular localization studies. *J Lipid Res*.2006;47(2) : 10.1194/jlr.M500321-JLR200 268-83 10.1194/jlr.M500321-JLR200 16269826
